# Estrogen Receptor (ER) and Progesterone Receptor (PgR) Expression in Endometrial Cancer—An Immunohistochemical Assessment

**DOI:** 10.3390/diagnostics14030322

**Published:** 2024-02-01

**Authors:** Stanisław Przewoźny, Jan Rogaliński, Mateusz de Mezer, Anna Markowska, Janina Markowska, Jakub Żurawski

**Affiliations:** 1 Department of Immunobiology, Poznan University of Medical Sciences, Rokietnicka 8, 60-806 Poznan, Poland; 2 Department of Perinatology and Women’s Diseases, Poznan University of Medical Sciences, Polna 33, 60-535 Poznan, Poland; 3 Gynecological Oncology Center, Poznańska 58A, 60-850 Poznan, Poland

**Keywords:** endometrial cancer, progesterone receptor, estrogen receptor, computer image analysis

## Abstract

Endometrial cancer (EC) is one of the most common types of cancer in Poland and worldwide. Many risk factors lead to the pathogenesis of this disease, such as lifestyle choices, BMI, the medicines used in breast cancer therapy, and Lynch syndrome. EC cells show the expression of estrogen receptors (ERs) and progesterone receptors (PgR). These receptors occur in multiple isoforms and have a significant influence on the operation of cells. The loss of ER and PgR expression is associated with a poor prognosis. We assessed tissue slides that were obtained from 103 women with EC diagnoses of various grades, stages, and histological types. In this study, we used computer image analyses to increase the objectivity of the assessment. We proved that, in the tissue of patients with high-grade (G3) EC, the expression of PgR is significantly lower than that in the tissues of patients with low-grade EC. We also observed that PgR is significantly expressed in EC with a low FIGO stage and in the endometroid type of EC (which rarely becomes malignant compared to serous type). The expression of ERb1 was lower in patients with EC at the IV FIGO stage than in patients with stage III EC. These findings confirm that the loss of ER and PgR expression is connected with a poor prognosis.

## 1. Introduction

The Polish National Cancer Register shows that there were 5286 cases of uterine corpus cancer reported in Poland in 2020, which is 7.11% of all cancers found in the country. Moreover, 1811 deaths caused by uterine corpus cancer were reported in 2020, which constitutes 3.98% of all cancer deaths reported in Poland that year [1]. One of the most common types of cancer in women in Poland and worldwide is endometrial cancer (EC) [2].

Lifestyle choices have a significant effect on the risk posed by endometrium cancer. A high BMI correlates with type I EC [3]. The risk factors for EC also include the following: a sedentary lifestyle, diabetes, metabolic syndrome, a low fertility rate, or meat consumption, especially red meat [4,5].

Type I EC is hormone-dependent, and its occurrence is influenced by the excessive stimulation of the endometrium by estrogen [3,6]. Tamoxifen, an estrogen-receptor-selective modulator that is used in breast cancer treatment, has an unfavorable influence on the endometrium and can lead to carcinogenesis [4].

The risk of endometrial cancer is also increased in women with Lynch syndrome (hereditary non-polyposis colorectal cancer) [7].

Endometrial cancer can be classified depending on its grade, stage, and histological type. The Bokhman classification includes two histological types: I—endometrioidal and II—non-endometrial; serous, clear cell, or low differentiated [8].

Type I EC is hormone-dependent and is of a low grade (G1/G2) and low FIGO stage (I-II). The mutation of the PTEN (phosphatase and tensin homologue), KRAS (Kirsten rat sarcoma virus homologue), CTNNB1 (β-catenin coding), PIK3CA (phosphatidylinositol-4,5-bisphosphate 3-kinase catalytic subunit alpha), and ARID1A (AT-Rich Interaction Domain 1A) genes and changes in the MSI (microsatellite instability) occur more often in type I EC than in type II EC. Type II EC is usually of a high grade and high FIGO stage. In type II EC, the mutation of the TP53 (tumor protein 53), HER2 (human epidermal growth factor 2), or PPP2R1A (protein phosphatase 2A) genes often occurs [8,9,10,11].

An alternative method used to subdivide endometrial cancer comprises an analysis of the variability in the polymerase e, MSI, p53 and L1CAM. These subtypes are as follows: POLE ultramutated; MSI hypermutated; copy number—high; and copy number—low [12,13].

### 1.1. Estrogen Receptors and Progesterone Receptors

**Figure 1:**
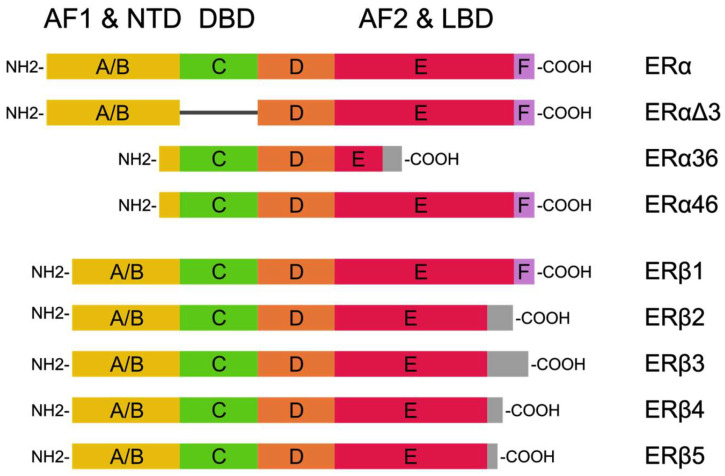
ER isoforms based on Pinton et al. [15] and Jia et al. [14]. Their structures consist of the N-terminal domain (NDT), the DNA-binding domain (DBD), and the C-terminal ligand-binding domain (LBD).

Estrogen receptors have two isoforms: ERα and ERβ. These isoforms are encoded by genes with different loci: ERα at 6q25.1, and ERβ at 14q23.2 [14]. Based on the length of the polypeptide chains and the weight of the proteins, these isoforms can be divided into ERαΔ3, ERα46, ERα36, ERβ1, ERβ2, ERβ3, ERβ4, and ERβ5 (Figure 1) [15]. Structurally, ER proteins consist of the following domains: the N-terminal, the DNA-binding domain, and C-terminal. The N-terminal domain is the most specific domain for each isoform (16% similarity between ERα and ERβ), and its structure includes the ligand-independent activation domain AF1 (which is involved in the transcriptional activation of target genes). The DNA-binding domain is an intermediate between the binding receptor and DNA in estrogen response elements (EREs); it is also the most conservative domain (97% similarity between ERα and ERβ). ERs also have an influence on the genes in EREs via the following transcription factors: AP-1, Sp1, and NF-κβ. The C-terminal domain exhibits a 59% similarity between ERα and ERβ, and its composition includes the ligand-dependent activating domain AF2 [14,16].

**Figure 2:**
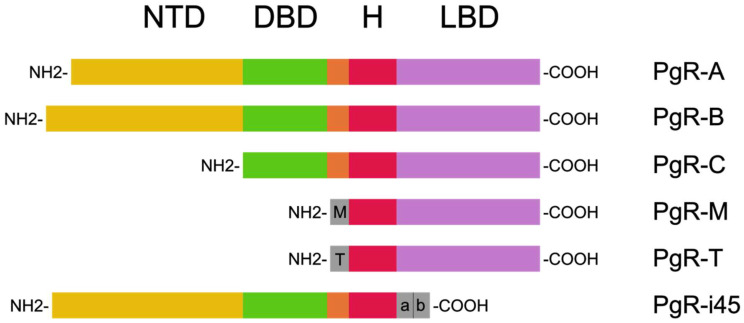
PgR isoforms based on Islam et al. [18]. These are composed of the N-terminal domain (NTD), the DNA-binding domain (DBD), the H region, and the ligand-binding domain (LBD).

Progesterone receptors can be found in the nuclear isoforms PgR-A and PgR-B, the mitochondrial isoform PgR-M, and other shorter isoforms such as PgR-C, PgR-T, and PgR-i45 (Figure 2). PgR can also exist in the form of progesterone receptor membrane components (PGRMC). The gene coding PgR-A and PgR-B is located in locus 11q22-q23, and the difference in its construction is a result of the transcription using different promoter sequences [17,18,19].

PgRs are nuclear receptors whose binding ligand exists within the cytoplasm. After binding with the PgR-A and PgR-B ligands, they first dissociate from the heat shock proteins and then relocate to the nucleus, where dimerization occurs. This complex in the nucleus binds with the transcription coactivators and progesterone response elements (PREs) within the target gene promoter, leading to the activation of transcription. The same complex binds with the corepressors within the target gene promoter and affects the suppression of transcription. In the case that the PREs leak, PgRs are still able to provide an indication of transcription; this is in cooperation with other DNA-binding transcription factors, namely AP-1, Sp1, STAT5, and NF-κβ [18].

The association between the loss of PgR and the expression of ERs with a poor prognosis in patients with EC has been shown [20]. This suggests that these changes correlate with the metastasis of lymph nodes and the risk of recurrence. Busch et al. found that there is an association between changes in the expression of ERs and PgR and improvements in the condition of treated patients [21].

### 1.2. The Aim of This Study

The purpose of this study was to analyze the expression of the estrogen receptors ERα and ERβ1 and progesterone (PgR) in endometrial cancers of the uterus using computer microscopic image analysis, as well as to evaluate the diagnostic value of the above parameters and the methodology used.

## 2. Materials and Methods

This study was approved by the Bioethics Committee of the Karol Marcinkowski University of Medical Science in Poznan. It was conducted in compliance with the Declaration of Helsinki. The Bioethics Committee’s statement of 16 January 2020 confirmed that the character of this study was not that of a medical experiment. This study did not need to be assessed by the Bioethics Committee, according to Good Clinical Practice (GCP) and Polish law. Each participant in the study gave informed consent.

The subjects of this experiment were 103 patients diagnosed with endometrium cancer (EC) in the years 2007–2014. Two of them (1.9%) were diagnosed with clear cell-type EC, fifteen (14.6%) were diagnosed with serous-type EC, and eighty six (83.5%) were diagnosed with endometrioidal-type EC. The histological EC type was assessed by pathologists according to the participants’ tissues and cell morphology. The grading distribution was as follows: G1—24 (23.3%), G2—37 (35.9%), and G3—42 (40.8%). In total, 49 patients (47.6%) were diagnosed with stage I EC; 23 patients (22.3%) were diagnosed with stage II EC, 16 patients (15.6%) were diagnosed with stage III EC, and 15 patients (14.6%) were diagnosed with stage IV EC. The EC stage was assessed based on the FIGO International Federation of Gynecologycal and Obstetrics system, which was updated in 2009.

Endometrial cancer tumors comprise cancer cells that form glandular tubules. Depending on the degree of differentiation and the cytological features observed, three grades can be distinguished:

Stage I—well-differentiated cancer. This is composed almost exclusively of glandular tubules. Solid fields do not constitute more than 5% of the weave.
Stage II—moderately differentiated cancer. Solid fields constitute 6% to 50% of the tumor tissue.
Stage III—poorly differentiated cancer. Solid fields occur in more than 50% of the tumor tissue. Greater mitotic activity and greater cell polymorphism are also observed.

The fragments of endometrium tissue that were collected during the surgical treatment were studied. The tissues were fixed in 10% formalin and then placed in paraffin blocks, which were used to make 5 μm thin scraps. Then, the scraps on the slides were deparaffinized and rehydrated in a range of alcohols, using distillated water at the end. Next, the slides were stained with hematoxylin for 5 min, flushed with tap water, and put into the water with a few drops of a saturated solution of lithium carbonate; this was performed until the nuclei turned blue. Then, the slides were stained with eosin for 1 min, flushed with distillated water, and dehydrated in a range of alcohols, using xylen at the end. The patients were diagnosed during the treatment process according to the hematoxylin–eosin-stained slides.

The slides used for the analyses were then immunohistochemically stained with the following specific antibodies: ERα (Santa Cruz Biotechnology, Santa Cruz, CA, USA sc-8005, clone D-12), ERβ1 (Zytomed Systems, Berlin, Germany MSK042-05, clone PPG5/10), and PgR (Dako, Santa Clara, CA, USA M3569, clone 636). For this purpose, the slides were incubated in a water bath heated to at 96 °C in citrate buffer at pH 6.0 for 50 min. Endogenous peroxidase was blocked using H_2_O_2_. In the next step, the preparations were incubated with antibodies for 60 min, after which they were washed in TBS buffer for 10 min. The slides were incubated for 30 min using the EnVision system (DakoCytomation, K5007; Dako, Santa Clara, CA, USA). In order to visualize the reaction, 3,3′-diaminobenzydin (DAB-3,3) was used. The last part of the preparation involved staining the slides with Mayers hematoxylin, performing dehydration and closing them with coverslips. An Olympus BX 43 microscope with a camera XC 30 (Olympus, Shinjuku, Tokyo, Japan) was used for the visual evaluation.

The morphometric analysis included the number of cells that were found to be immunopositive for all tested receptors that were detected in the immunohistochemical reactions: ERα, ERβ1, and PgR. Six images of the field of view were taken for each sample, at a total magnification of 400×. For every slide, areas with immunopositive cells were chosen. The desmoplasia area was included in the analysis if it was found in the immediate vicinity of the assessed region. The evaluation fields of the slides were obtained by moving the stage knob a ¼ turn along axis X or axis Y. Therefore, the creation of double pictures from one slide was prevented. Areas that exhibited necrosis or artifacts were excluded from the morphometric analysis. For this purpose, an Olympus BX 43 light microscope and an XC 30 digital camera were used. The phase analysis of the stained preparation was performed according to a program that performed the automatic detection of objects based on their color (brown chromogen DAB-3.3). Based on the established threshold values, automatic classification was performed using software. The microscope had been previously calibrated using a computer program. The obtained surface area values were expressed in μm^2^ Early malignant changes were absent in the women subjected to Aquafilling breast augmentation that was based on the E-cadherin and N-cadherin immunohistochemical expression [22]. 

The positive control used was an exact internal control for the immunohistochemical reaction. Similarly, the positive control used was the internal control for the immunohistochemical reaction. The slides without primary antibodies were used as the negative control.

The data obtained were subjected to statistical analysis. With regard to the clinical characteristics, the level of each marker between the subjects of the groups were compared, depending on the normality of the distributions (which was evaluated with the Shapiro–Wilk test). The mean, standard deviations or median; quartiles 1 and 3; and the range of results (minimum and maximum) for all distributions were obtained. For comparisons, both parametric and non-parametric tests were used: the Mann–Whitney U test or Student’s *t*-test was used for the comparison of two groups, and the Kruskal–Wallis test or ANOVA was used for the comparison of more than two groups. Post hoc analyses (conducted for significant Kruskal–Wallis test results) were performed according to Tukey’s method. The analysis assumed significance at a level of α = 0.05, and calculations were performed with the help of the R statistical software, version 4.2.1.

## 3. Results

**Figure 3:**
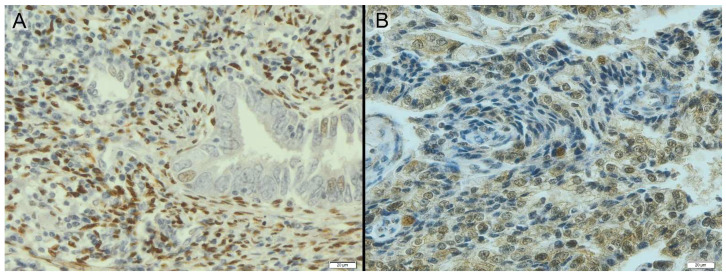
Endometrium with positive adenocarcinoma diagnosis stained immunohistochemically for PgR (**A**) and ERβ (**B**)—brown color.

The patients with clinical stage IV EC were characterized by significantly lower levels of the ERβ1 variable than those with clinical stage III EC (*p* = 0.005 for the analysis comparing all four groups, and *p* = 0.002 for the post hoc analysis) (Figure 3 and Table 1).

The patients with histological grade G3 EC had significantly lower levels of the PgR variable than the other two groups (*p* = 0.003 for the analysis comparing all three groups, *p* = 0.032 for G1 vs. G3, and *p* = 0.005 for G2 vs. G3). The clinical stage of EC also differed with regard to the level of the PgR variable among the patients (*p* = 0.008 for the comparison of all four groups). Patients with stage I EC had significantly higher levels of the PgR variable than those with clinical stage II and IV EC (*p* = 0.028 for I vs. II and *p* = 0.035 for I vs. IV).

Significantly higher levels of the PgR variable were observed among the patients with endometrioid adenocarcinoma than among those with serous adenocarcinoma (*p* = 0.029). Other analyses and post hoc comparisons were statistically insignificant (*p* > 0.050).

## 4. Discussion

In physiology, the higher expression of ERα occurs at the proliferative phase of the menstrual cycle. It upregulates the proliferation, migration, and angiogenesis of cells, and inhibits their apoptosis [16]. In early-stage EC, higher ERα expression has been observed [23].

Hapangama et al. concluded that ERβ plays an important role in the physical functions of the endometrium, so the lower expression of ERβ observed in our study is in accordance with these findings [24]. ERα and ERβ can act antagonistically: ERα promotes cell growth, while ERβ stimulates cell cycle progression and the expression of apoptosis genes [15]. Moreover, ERβ can inhibit ERα’s proliferative properties [14]. Wang et al. showed that the total pool of estrogen receptors decreases as the clinical stage increases [25]. These circumstances suggest that the downregulation of ERb1 expression we observed in stage IV EC is justified. The lack of a proper cell cycle guardian protein can promote the development of cancer. However, other studies have suggested that the expression of ERβ is upregulated in higher cancer stages. However, these authors differentiated the patients into two groups: patients exhibiting the IA FIGO stage and others exhibiting higher stages of EC; this narrows the interpretation of the results [23].

Despite the structural similarities in the DNA-binding domain, ERα and ERβ can affect the transcription of different gene sets, and can even operate antagonistically. The high expression of ERα correlates with the high expression of genes associated with proliferation. ERβ favors cell cycle progression and the expression of apoptosis genes. In addition, ERβ can inhibit 70% of the genes regulated by ERα. The regulation of ER transcription also takes place through gene coregulators. Coregulator sets differ significantly between isoforms α and β [15,16].

The role of PgR in carcinogenesis is not as clear. Wilson et al. suggest that the expression of PgR may be indicated by ARID1A and downregulation accompanied by EZH2 mediation [26]. Also, DNA methylation can influence PgR regulation [27]. It has been proven that the isoform PgR-A is able to block the transcription activity of ERs [28].

Consistent with our results, Arnett-Mansfield et al. found that PgR expression is downregulated in late-stage EC [29]. Reid-Nicholson et al. found lower PgR expression in grade III EC compared to lower-grade EC [30].

It is likely that the loss of PgR expression is a consequence of cancerous transformation. A lower PgR expression is connected with an increase in the relative abundance of estrogen receptors, which promote proliferation. In addition, studies have shown that the downregulation of PgR expression occurs in tumors, but in hyperplastic cells, the level of PgR expression is not significantly different from regular cells [29].

In our study, we observed significantly higher PgR expression in endometroid-type EC than serous-type EC. Markowska et al. noted similar results, with a higher expression of PgR in type I EC than type II EC [2]. The downregulated expression of PgR in serous-type EC may be connected with cell nuclei changes. The nucleus of endometroid-type EC compared with that of serous-type EC is moderately atypical, and metaplasia can sometimes be observed [10]. The nucleus of serous-type EC is clearly atypical, and is often pleomorphic, hyperchromic, and includes macronucleoli [31,32]. Studies have not only shown the lower expression of PgR in serous-type EC, but also the loss of ERs [33].

**Figure 4:**
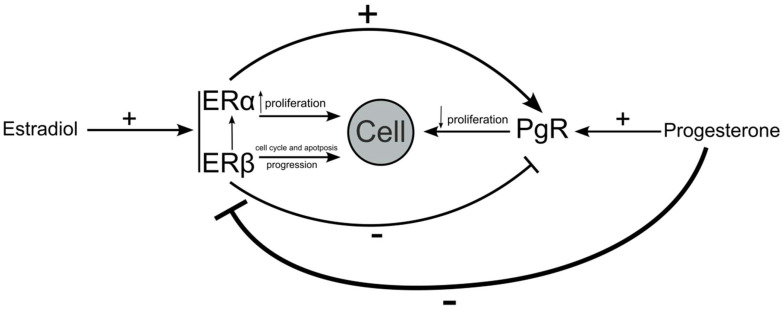
Dependence of estrogen and progesterone receptors on each other. Estradiol stimulates ERα and Erβ; this is in addition to their effects on cell regulation and the expression of PgR. PgR is stimulated by progesterone, which, in addition to affecting cell metabolism, also decreases the expression of estrogen receptors.

Studies have shown that estradiol may affect the expression of PgR via the expression of ERs. PgR genes include palindromic EREs [34]. EREs act as an anchor for ERα and help to recruit transcription factors [35]. This results in the upregulation of PgR expression. Interestingly, the effects that ERβ has on the expression of PgR are opposite to those of ERα. Stimulated ERβ has the ability to downregulate the expression of mRNA and the PgR protein [16]. In conclusion, the proteins we evaluated in this study are highly interdependent. The presence and functionality of estrogen receptors determine the effect that estradiol has on cells; in addition to the fact that ER isoforms compete with each other, estradiol affects the ability of ERs to regulate the expression of PgR. The increase or decrease in PgR expression therefore depends on the ERα:ERβ ratio (Figure 4).

Our results have been confirmed by many of the researchers we have mentioned in this study, despite occasional differences in the conclusions drawn. However, in this study, we used an alternate research method and confirmed its effectiveness. Furthermore, the computer microscopic image analysis employed was faster and less tiring for the researcher. The unparalleled advantage of this method is that it reduces the negative impact of less experienced researchers performing a subjective evaluation. In conclusion, using computer microscopic image analysis in immunohistochemistry enables more slides to be assessed more quickly and objectively.

## 5. Conclusions

1. The downregulated expression of ERβ1 in the tissue of patients diagnosed with EC could suggest grade III cancer;
2. The downregulated expression of PgR in the tissue of patients diagnosed with EC could suggest grade III cancer. Meanwhile, a high expression of this receptor can indicate stage I stage or Adenocarcinoma endometroides;
3. PgR can be a good prognostic and diagnostic tool when aiming to determine the EC grade, its prognosis, and its histological type;
4. Computer microscopic image analysis ensures that the assessment is objective and reduces the time required for analysis.

## Figures and Tables

**Table 1:** M—mean; SD—standard deviations; Me—median; Q1, Q3—quartile 1 and 3. The adenocarcinoma clarocellulare group was not included in the histological diagnosis due to an insufficient number of patients (*n* = 2). For analyses, the Mann–Whitney U test or Student’s *t*-test was used when comparing two groups, and the Kruskal–Wallis test was used when comparing more groups. Statistically significant results are in bold.

Clinical Characteristics	M ± SD/Me (Q1; Q3)	*P*
	ERα	
Grading		
G1	2217.16 (818.47; 4118.53)	0.538
G2	2080.48 (508.98; 3696.69)	
G3	1781.10 (180.61; 3102.98)	
FIGO staging		
I	2063.94 (922.12; 3919.11)	0.133
II	919.42 (44.10; 2288.30)	
III	2486.85 (732.53; 4273.29)	
IV	1767.39 (262.35; 3902.94)	
Histological type		
*Adenocarcinoma endometroides*	1977.05 (521.86; 3692.75)	0.596
*Adenocarcinoma serous*	2251.14 (110.75; 3510.44)	
	ERβ1	
Grading		
G1	1368.50 (319.29; 2623.64)	0.948
G2	1652.46 (243.74; 4276.30)	
G3	1134.74 (240.61; 4816.59)	
FIGO staging		
I	1293.68 (243.74; 3027.34)	0.005
II	1109.56 (377.35; 4201.59)	
III	4798.04 (2267.15; 7489.39)	
IV	285.46 (64.20; 1752.23)	
Histological type		
*Adenocarcinoma endometroides*	1698.21 (288.45; 4714.87)	0.231
*Adenocarcinoma serous*	861.08 (191.28; 2541.03)	
	PgR	
Grading		
G1	10,316.56 (8239.62; 12,331.57)	0.003
G2	9981.14 (6659.41; 13,416.16)	
G3	5289.84 (2235.35; 10,221.28)	
FIGO staging		
I	10,566.50 (7148.94; 13,438.49)	0.008
II	4976.73 (2660.06; 10,181.67)	
III	9463.98 (6578.57; 12,562.79)	
IV	6866.14 (3150.23; 7986.22)	
Histological type		
*Adenocarcinoma endometroides*	9577.68 ± 5024.92	0.029
*Adenocarcinoma serous*	6110.82 ± 5258.12	

## Data Availability

The raw data supporting the conclusions of this article will be made available by the authors on request.

## References

[1] https://onkologia.org.pl/pl/raporty.

[2] Markowska A., Grybos A., Marszałek A., Bednarek W., Filas V., Gryboś M., Markowska J., Mądry R., Więckowska B., Nowalińska D. (2021). Expression of selected molecular factors in two types of endometrial cancer. Adv. Clin. Exp. Med..

[3] Setiawan V.W., Yang H.P., Pike M.C., McCann S.E., Yu H., Xiang Y.-B., Wolk A., Wentzensen N., Weiss N.S., Webb P.M. (2013). Type I and II Endometrial Cancers: Have They Different Risk Factors?. J. Clin. Oncol. Off. J. Am. Soc. Clin..

[4] Raglan O., Kalliala I., Markozannes G., Cividini S., Gunter M.J., Nautiyal J., Gabra H., Paraskevaidis E., Martin-Hirsch P., Tsilidis K.K. (2019). Risk factors for endometrial cancer: An umbrella review of the literature. Int. J. Cancer.

[5] Ali A.T. (2014). Reproductive Factors and the Risk of Endometrial Cancer. Int. J. Gynecol. Cancer Off. J. Int. Gynecol. Cancer Soc..

[6] Yeramian A., Moreno-Bueno G., Dolcet X., Catasus L., Abal M., Colas E., Reventos J., Palacios J., Prat J., Matias-Guiu X. (2013). Endometrial carcinoma: Molecular alterations involved in tumor development and progression. Oncogene.

[7] Lu K.H., Daniels M. (2013). Endometrial and ovarian cancer in women with Lynch syndrome: Update in screening and prevention. Fam. Cancer.

[8] Wilczyński M., Danielska J., Wilczyński J. (2016). An update of the classical Bokhman’s dualistic model of endometrial cancer. Menopausal Rev..

[9] Goebel E.A., Vidal A., Matias-Guiu X., Gilks C.B. (2018). The evolution of endometrial carcinoma classification through application of immunohistochemistry and molecular diagnostics: Past, present and future. Virchows Arch..

[10] Murali R., Davidson B., Fadare O., Carlson J.A., Crum C.P., Gilks C.B., Irving J.A., Malpica A., Matias-Guiu X., McCluggage W.G. (2019). High-grade Endometrial Carcinomas: Morphologic and Immunohistochemical Features, Diagnostic Challenges and Recommendations. Int. J. Gynecol. Pathol..

[11] Koskas M., Amant F., Mirza M.R., Creutzberg C.L. (2021). Cancer of the corpus uteri: 2021 update. Int. J. Gynecol. Obstet..

[12] Chang Z., Talukdar S., Mullany S.A., Winterhoff B. (2019). Molecular characterization of endometrial cancer and therapeutic implications. Curr. Opin. Obstet. Gynecol..

[13] Arciuolo D., Travaglino A., Raffone A., Raimondo D., Santoro A., Russo D., Varricchio S., Casadio P., Inzani F., Seracchioli R. (2022). TCGA Molecular Prognostic Groups of Endometrial Carcinoma: Current Knowledge and Future Perspectives. Int. J. Mol. Sci..

[14] Jia M., Dahlman-Wright K., Gustafsson J.Å. (2015). Estrogen receptor alpha and beta in health and disease. Best Pract. Res. Clin. Endocrinol. Metab..

[15] Pinton G., Manzotti B., Balzano C., Moro L. (2021). Expression and clinical implications of estrogen receptors in thoracic malignancies: A narrative review. J. Thorac. Dis..

[16] Yu K., Huang Z.-Y., Xu X.-L., Li J., Fu X.-W., Deng S.-L. (2022). Estrogen Receptor Function: Impact on the Human Endometrium. Front. Endocrinol..

[17] Reis F.M., Coutinho L.M., Vannuccini S., Batteux F., Chapron C., Petraglia F. (2020). Progesterone receptor ligands for the treatment of endometriosis: The mechanisms behind therapeutic success and failure. Hum. Reprod. Updat..

[18] Islam S., Afrin S., Jones S.I., Segars J. (2020). Selective Progesterone Receptor Modulators—Mechanisms and Therapeutic Utility. Endocr. Rev..

[19] Wu S.-P., DeMayo F.J. (2017). Progesterone Receptor Signaling in Uterine Myometrial Physiology and Preterm Birth. Curr. Top. Dev. Biol..

[20] Smith D., Stewart C.J., Clarke E.M., Lose F., Davies C., Armes J., Obermair A., Brennan D., Webb P.M., Nagle C.M. (2018). ER and PR expression and survival after endometrial cancer. Gynecol. Oncol..

[21] Busch E.L., Crous-Bou M., Prescott J., Chen M.M., Downing M.J., Rosner B.A., Mutter G.L., De Vivo I. (2017). Endometrial Cancer Risk Factors, Hormone Receptors, and Mortality Prediction. Cancer Epidemiol. Biomarkers Prev..

[22] Chalcarz M., Żurawski J. (2022). The absence of early malignant changes in women subjected to Aquafilling breast augmentation on the basis of E-cadherin and N-cadherin immunohistochemical expression. Cent. Eur. J. Immunology..

[23] Markowska A., Szarszewska M., Żurawski J., Sajdak S., Knapp P., Gryboś A., Oleje A., Bednarek W., Roszak A., Jóżwik M. (2018). Studies on selected molecular factors in endometrial cancers. Adv. Clin. Exp. Med..

[24] Hapangama D., Kamal A., Bulmer J. (2015). Estrogen receptor β: The guardian of the endometrium. Hum. Reprod. Updat..

[25] Wang C., Tran D.A., Fu M.Z., Chen W., Fu S.W., Li X. (2020). Estrogen Receptor, Progesterone Receptor, and HER2 Receptor Markers in Endometrial Cancer. J. Cancer.

[26] Wilson M.R., Reske J.J., Koeman J., Adams M., Joshi N.R., Fazleabas A.T., Chandler R.L. (2022). SWI/SNF Antagonism of PRC2 Mediates Estrogen-Induced Progesterone Receptor Expression. Cells.

[27] Wu Y., Strawn E., Basir Z., Halverson G., Guo S.-W. (2006). Promoter Hypermethylation of Progesterone Receptor Isoform B (PR-B) in Endometriosis. Epigenetics.

[28] Wen D.X., Xu Y.F., E Mais D., E Goldman M., McDonnell D.P. (1994). The A and B isoforms of the human progesterone receptor operate through distinct signaling pathways within target cells. Mol. Cell. Biol..

[29] Arnett-Mansfield R.L., de Fazio A., Wain G.V., Jaworski R.C., Byth K., Mote P.A., Clarke C.L. (2001). Relative expression of progesterone receptors A and B in endometrioid cancers of the endometrium. Cancer Res..

[30] Reid-Nicholson M., Iyengar P., Hummer A.J., Linkov I., Asher M., A Soslow R. (2006). Immunophenotypic diversity of endometrial adenocarcinomas: Implications for differential diagnosis. Mod. Pathol..

[31] Clement P.B., Young R.H. (2004). Non-Endometrioid Carcinomas of the Uterine Corpus: A Review of Their Pathology With Emphasis on Recent Advances and Problematic Aspects. Adv. Anat. Pathol..

[32] A Soslow R. (2012). High-grade endometrial carcinomas–strategies for typing. Histopathology.

[33] Bartosch C., Lopes J.M., Oliva E. (2011). Endometrial Carcinomas: A Review Emphasizing Overlapping and Distinctive Morphological and Immunohistochemical Features. Adv. Anat. Pathol..

[34] Pu H., Wen X., Luo D., Guo Z. (2022). Regulation of progesterone receptor expression in endometriosis, endometrial cancer, and breast cancer by estrogen, polymorphisms, transcription factors, epigenetic alterations, and ubiquitin-proteasome system. J. Steroid Biochem. Mol. Biol..

[35] Petz L.N., Ziegler Y.S., Schultz J.R., Kim H., Kemper J., Nardulli A.M. (2004). Differential regulation of the human progesterone receptor gene through an estrogen response element half site and Sp1 sites. J. Steroid Biochem. Mol. Biol..

